# Eighth Annual Meeting of the American Society of Dental Surgeons

**Published:** 1847-06

**Authors:** 


### EIGHTH ANNUAL MEETING OF THE AMERICAN SOCIETY OF DENTAL SURGEONS.

We would remind the members of the American Society of Dental Surgeons, and such others of our readers that feel an interest in the convocations of this association, that its eighth annual meeting will be held at Saratoga Springs, on the first Tuesday of August next. We do not feel it necessary to urge upon any of its members the importance of attendance, as the unfinished business of the last meeting has a most direct bearing upon the prosperity of the association, if not the individual interest of its members. And in this connection, we would express our earnest wish for an *early* adjustment of the question which has heretofore materially embarrassed and retarded the business of the association; and we trust not a few will be gratified to learn that the present indications bid fair that such a wish will be realized.

The association has been in existence a sufficient period to test its stability, and has attained sufficient maturity and strength to be classed with the scientific and influential organizations of our country, and it needs not prophetic powers to determine its influence, if its deliberations and doings are guided by prudence, liberality, and a single eye to the designs and principles of the society.

The association is designed as a national institution, administering to the interests of the most distant and remote of its members; but to make it effective and useful to its full extent, these interests must be represented by its members, and the doings of its meetings free from local favoritism.

It is to be hoped that the association will not omit to set apart, as sacred, a portion of time for oral discussion and exchange of opinions. It may be expected the society will take some steps in establishing a code of ethics, and carry out the measures proposed for accumulating the statistical results of the experience and observations of its members.

We cannot but think that a large number of the members of the society will be present, as few of the profession can find an excuse for absence, where there is so much, in point of location and interest to invite their attendance.

